# Bilateral sympathetic stellate ganglionectomy attenuates myocardial remodelling and fibrosis in a rat model of chronic volume overload

**DOI:** 10.1111/jcmm.14000

**Published:** 2018-11-08

**Authors:** Mingjing Zhang, Pengfei Zhu, Yuting Wang, Jie Wu, Yijun Yu, Xinying Wu, Xiaoyan Liu, Ye Gu

**Affiliations:** ^1^ Wuhan Fourth Hospital Puai Hospital Tongji Medical College Huazhong University of Science and Technology Wuhan China

**Keywords:** bilateral sympathetic stellate ganglionectomy, cardiac remodelling, partial cardiac sympathetic denervation, sympathetic neurohormones

## Abstract

Reducing sympathetic neurohormone expression is a key therapeutic option in attenuating cardiac remodelling. Present study tested the feasibility of attenuating cardiac remodelling through reducing sympathetic neurohormone level by partial cardiac sympathetic denervation in a rat model of chronic volume overload. Male Sprague‐Dawley rats were randomized into sham group (S, n = 7), aortocaval fistula group (AV, n = 7), and aortocaval fistula with bilateral sympathetic stellate ganglionectomy group (AD, n = 8). After 12 weeks, myocardial protein expression of sympathetic neurohormones, including tyrosine hydroxylase, neuropeptide Y, growth associated protein 43, and protein gene product 9.5, were significantly up‐regulated in AV group compared to S group, and down‐regulated in AD group. Cardiac remodelling was aggravated in AV group compared to S group and attenuated in AD group. The myocardial deposition of extracellular matrix, including collagen I and III, was enhanced in AV group, which was reduced in AD group. Myocardial angiotensin II and aldosterone expressions were significantly up‐regulated in AV group and down‐regulated in AD group. Our results show that bilateral sympathetic stellate ganglionectomy could attenuate cardiac remodelling and fibrosis by down‐regulating sympathetic neurohormones expression in this rat model of chronic volume overload.

## 1 INTRODUCTION

Heart failure is viewed as a progressive disease process, represented by cardiac dysfunction and dyspnea, which is usually initiated after an “index event,” as in the case of volume overloading 1 or myocardial infarction.^2^ As a compensatory process, sympathetic nervous system (SNS) is steadily activated to sustain cardiac output in response to impaired cardiac pumping and/or filling properties.^3^ However, sustained activation of SNS would inflict secondary damage on failing hearts and aggravate the cardiac remodelling and dysfunction.^1^, ^4^ It is known that heart failure is characterized by vagal attenuation and, over time, progressively pathological augmentation of sympathetic out‐flow, this sympathetic/parasympathetic imbalance occurs steadily irrespective of the etiology of heart failure.^5^, ^6^, ^7^ Modalities, including strategies modulating the autonomic nervous system, are intensively searched to correct this dangerous “autonomic imbalance” in the setting of heart failure.^5^ Previous studies demonstrated that drug therapy aimed to block the SNS and renin–angiotensin–aldosterone systems (RAAS) consistently reduced morbidity and mortality in chronic heart failure patients with reduced LV ejection fraction.^8^ Besides β‐blocker, researchers also explored other strategies to reduce the secondary damage induced by excessive release of sympathetic hormones on the failing heart.^9^, ^10^ One promising option is the blockade or removal of sympathetic ganglion. It was shown that surgical removal of sympathetic ganglion via ganglionectomy could denervate the heart and reduce the activity of cardiac sympathetic neurohormones including norepinephrine, tyrosine hydroxylase (TH), and neuropeptide Y (NPY) etc.^11^ Neurohormone TH, NPY, growth associated protein 43 (GAP43), and protein gene product 9.5 (PGP9.5) were the biomarkers of SNS, the neurohormone level could reflect SNS activity. Previous studies showed that, TH served as a sympathetic nerve marker. NPY was the neurotransmitter substance in neurons and postulated to be responsible for sympathovagal crosstalk. GAP43 played a crucial role in neural development, axonal regeneration, and structural plasticity. Panneuronal marker PGP9.5 was the marker of neuron fiber, which indicated the function of the autonomic nervous system.^11^, ^12^, ^13^, ^14^ Schwartz and colleagues demonstrated the beneficial effect of cardiac sympathetic denervation on suppressing ventricular tachycardia (VT) and fibrillation (VF) in patient refractory to VT ablation.^15^, ^16^, ^17^ Similarly, Shivkumar and associates also showed that bilateral cardiac sympathetic denervation was an effective therapeutic strategy to treat patients with VT, VF, and electrical storm.^18^, ^19^, ^20^ Above studies thus indicated that cardiac denervation via sympathectomy is a clinically feasible strategy to partial denervate the heart and treat patients with malignant ventricular arrhythmias. Results from these studies also hint the possibility to alleviate the secondary damage of excessive release of sympathetic hormones on the failing heart by means of cardiac sympathetic denervation. In fact, beneficial effects had been observed in patients with chronic heart failure treated with left cardiac sympathetic denervation.^21^ A prospective clinical study is now underway to assess the effect of left cardiac sympathetic denervation in patients with chronic heart failure.^22^

Till now, the effect and mechanism of ganglionectomy on experimental heart failure models are not fully understood. Previous clinical studies demonstrated that bilateral sympathectomy was a preferred option for the treatment of refractory ventricular tachycardia versus left sympathectomy, as the efficacy of left sympathectomy alone on preventing or reducing the incidence of malignant ventricular arrthymias was sometimes suboptimal.^20^, ^23^, ^24^ Symmetric distribution of sympathetic nerve in left and right heart belonged to the usual biological feature, bilateral sympathetic stellate ganglionectomy (SGX) might thus be helpful to re‐establish the sympathetic balance at lower level, we thus chosen the bilateral SGX strategy as a tool to partially reduce the overactivated sympathetic activity in the failing heart. In the current study, we tested the hypothesis that bilateral SGX surgery might be an effective strategy for the partial denervating of the heart via lowering the expression of cardiac sympathetic neurohormones, and subsequently attenuating the cardiac remodelling in a rat model of sustained volume overload.

## 2 MATERIALS AND METHODS

### 2.1 Animal preparation and study protocol

Male Sprague‐Dawley (SD) rats (180‐200 g) were purchased from the Experimental Animal Center of Tongji Medical College of Huazhong University of Science and Technology. All procedures were approved by the Animal Care Committee of Huazhong University of Science and Technology. Animals were maintained in accordance with the Guide for the Care and Use of Laboratory Animals published by the United States National Institute of Health (NIH Publication, 8th Edition, 2011). All surgeries were performed under sodium pentobarbital anaesthesia, and efforts were made to minimize the suffering of experimental animals. Rats were housed under standard conditions with free access to food and drinking water. Rats received a normal salt diet (0.3% NaCl) throughout the study.

The first surgery was performed to establish the aortocaval fistula (AV) model in anaesthetized (40 mg/kg sodium pentobarbital intraperitoneally) rats. Ventral abdominal laparotomy was performed in anaesthetized rats, the intestines were displaced laterally and wrapped with normal saline‐soaked sterilized gauze to retain moisture. The aorta and vena cava between the levels of renal arteries and iliac bifurcation were then exposed by blunt dissection of the overlaying adventitia. Both vessels were temporarily occluded proximal and distal to the intended puncture site, and a 18‐gauge needle held on a plastic syringe was inserted into the exposed abdominal aorta and advanced through the medial wall into the vena cava to create the shunt. The needle was gently inserted and withdrawn across the medial wall several times through the same puncture hole, to ensure the establishment of the fistula, before the needle was finally withdrawn from the aorta. The ventral aortic puncture site was immediately sealed with a drop of cyanoacrylate (Medical Adhesive Glue; Baiyun Medical Adhesive Co., Guangzhou, China) after withdrawal of the needle. Creation of a successful fistula was confirmed by visualizing the pulsatile flow of oxygenated blood into the vena cava from the abdominal aorta. The intestines were repositioned, and the abdominal musculature and skin incisions were closed by standard techniques with absorbable suture and autoclips.^25^

### 2.2 Stellate ganglionectomy surgery

The partial denervation of the cardiac sympathetic nerves was accomplished by bilateral SGX. It was technically difficult to perform the surgical removal of bilateral stellate ganglion at one operation session. To improve the survival of rats assigned for SGX, we choose to achieve the bilateral SGX at two separate operative sessions at 1‐week interval (at 7th and 14th days post AV).

**Figure 1:**
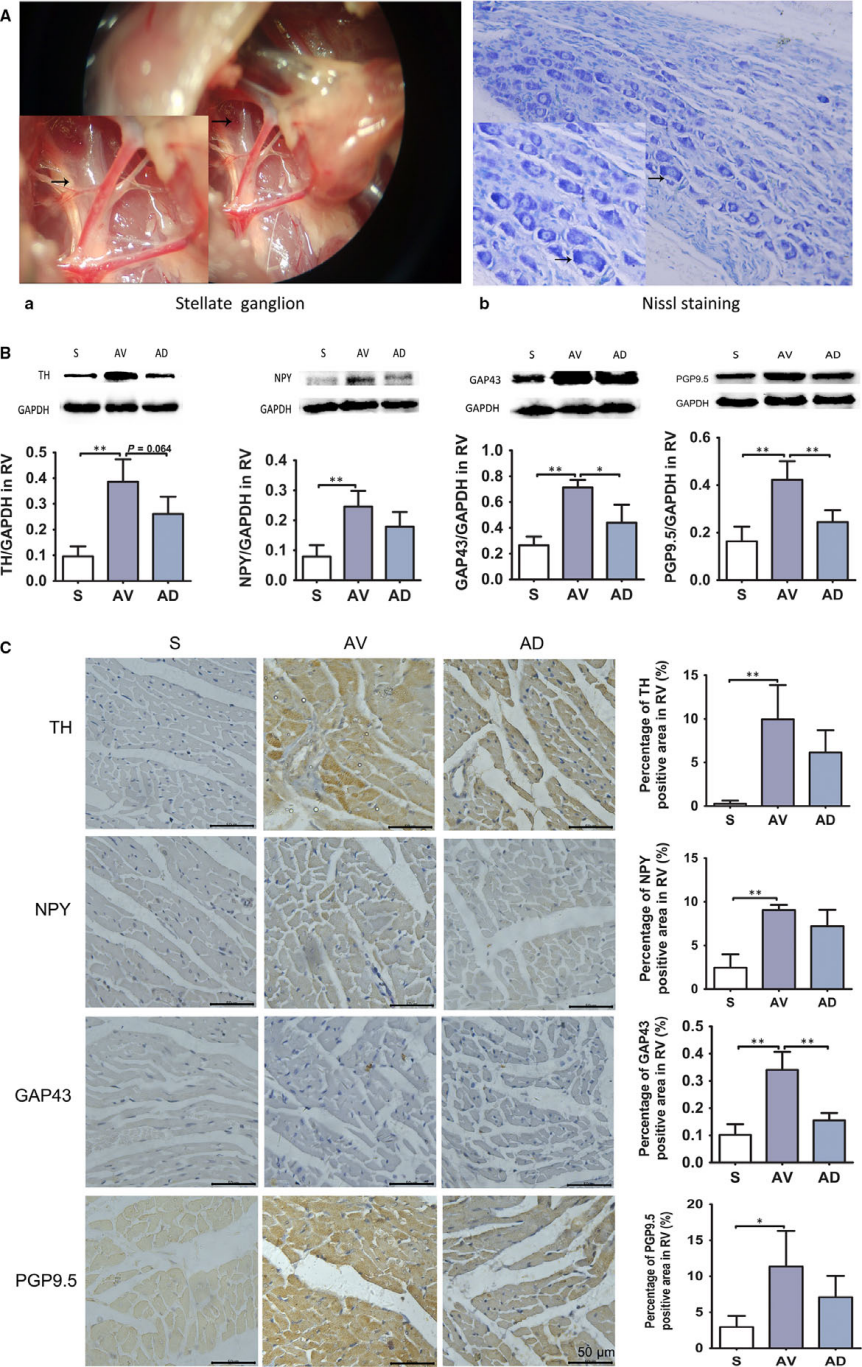
A, The stellate ganglion was exposed under the surgical microscope. All nerve branches running into the ganglion and the ganglion were isolated and excised (a, ×10). The ganglion was processed for paraffin embedding and sliced into 4 μm tissue sections, stained blue with Nissl staining (Arrow) (b, ×200). B, Protein expression of sympathetic neurohormones in RV. Protein expression of TH, NPY, GAP43 and PGP9.5 in RV were up‐regulated in AV group (vs S group) and protein expression of GAP43 and PGP9.5 was down‐regulated in AD group (vs AV group) (*P <* *0.05,**P <* *0.01). (S, AV and AD, n = 4). C, Immunohistochemistry staining of sympathetic neurohormones in RV. The protein expression of TH, NPY, GAP43 (**P <* *0.01) and PGP9.5 (*P <* *0.05) was increased in AV group (vs S group) and protein expression of GAP43 (**P <* *0.01) was reduced in AD group compared to AV group in RV (×400). (S, AV and AD, n = 3)

Seven days post AV operation, rats were re‐anaesthetized, intubated endotracheally using a 16 G intravenous catheter connected to a ALCOTT rodent ventilator (model ALC‐V8S, China). The ventilator was set at 65 cycles/min and 2.5 mL stroke volume. Following a right sided skin incision, thoracotomy was performed by cutting the second rib on the right side. The incision was retracted, and the right lung was gently pulled caudally using sterile saline‐soaked gauze. A surgical microscope (model JSZ6, RWD, China) was used to visualize the right stellate ganglion between the first and second ribs beneath the parietal pleura (Figure 1A‐a). All nerve branches running into the ganglion were isolated and cut, the ganglion was excised, and the gauze was removed. The removed stellate ganglion was stained with Nissl staining (Figure 1A‐b). A left SGX was performed using the same procedure on the left side 1 week later. The thorax was closed after each operation session. A negative intrathoracic pressure was re‐established by inserting a 23‐gauge needle into the thoracic cavity and suctioning air with a 3‐ml syringe. All rats underwent SGX exhibited ptosis the day after surgery, an initial indicator of a successful ganglionectomy.^26^

### 2.3 Echocardiography examination

Echocardiography examination was performed at 12 weeks post‐Sham or AV operation by a researcher blinded to the study protocol. Left parasternal and left apical echocardiographic images of anaesthetized rats lying in a supine position were obtained with an echocardiographic system (GE Vivid 7) equipped with a 11.4 MHz transducer. A two‐dimensional short‐axis view of the left ventricle was obtained at the level of the papillary muscles. Left ventricular measurements including the end‐diastolic and end‐systolic internal dimension (LVIDd and LVIDs, mm), ejection fraction (EF, %), and fractional shortening (FS, %) were measured.

### 2.4 Blood and tissue collection and biochemistry examination

After echocardiographic examination and blood sampling from vena cava, the rats were killed under additional deep anaesthesia. After washing out the blood of the rat hearts, heart from three randomly selected rats from each group was longitudinally divided into two portions, one portion for gross morphology, another portion for histological examinations. The remaining four hearts of S and AV group, and five hearts of AD group were used for the measurement of total heart weight, then the hearts were divided into atria, right ventricular (RV) and left ventricular (LV) including the septum parts and each part was weighed, then frozen at −80°C for future molecular biological examinations. One portion longitudinal three hearts which randomly selected from each groups were embedded in paraffin. Tissue sections were stained with haematoxylin‐eosin (HE), Sirius red staining and immunohistochemistry methods, respectively, and images were then captured with a Leica microscope (DMi8; Wetzlar, Germany). Another portion longitudinal three hearts were fixed in neutral formaldehyde for 1 week, the hearts were then blotted dry to present the size of the cavity and thickness of the RV and LV walls. Angiotensin II (Ang II) concentrations in the plasma was measured with the ELISA kit (RayBio, EIA‐ANG II, Norcross, GA, USA) following manufacturer's instructions. The absorbance was recorded at 450 nm.

### 2.5 Pathology examination

HE, Sirius red staining, and immunohistochemistry staining were performed on heart tissue sections, image was captured by microscope (DMi8; Leica, Germany) with 400 magnification. Cardiomyocyte size was determined by measuring the cardiomyocyte cross‐sectional area (μm^2^), 150 cross‐sectioned cardiomyocytes were counted in at least 10 images obtained from left and right ventricle per rat, respectively, on HE stained section.^27^ Manual tracing of the cardiomyocyte cross‐sectional area was performed with Image ProPlus (IPP) (Media Cybernetics, Inc., Rockville, MD, USA), at 400X magnification. The cardiomyocyte cross‐sectional area was measured per nucleus, and only cardiomyocyte that were cut in the same direction were included in the measurement.^28^, ^29^ Extracellular matrix of collagen was measured on Sirius red stained tissue samples, the mean values were evaluated by assessing of percent of collagen in 15 randomly captured pictures of left and right ventricle, respectively. IPP was used as the tool of measure, under Count/Size window, selecting collagen with red colour by tubularis, the sum IOD of collagen was obtained. For immunohistochemistry staining, three‐step technique (Histostain‐Plus IHC Kit, NeoBioscience, Shenzhen, China) was used for visualization. Primary antibodies for immunohistochemistry were as follow: TH (1:300, ab137869; Abcam, Cambridge, UK), NPY (1:500, ab30914; Abcam), GAP43 (1:100, ab117265; Abcam), PGP9.5 (1: 500, ab8189; Abcam), Ang II (1:100, GTX37789; GeneTex, CA, USA) and aldosterone (1:1000, ARG10693; Arigo, Taiwan, China). IPP was used as the tool of measure, under Count/Size window, selecting positive expression with brown colour by tubularis, the sum IOD of positive expression was obtained. The values of 15 randomly captured pictures per rat of left and right ventricle were averaged. The immunohistochemical densitometry was ratioed to background for consistency between slides before picture capture. The paraffin sections were deparaffinized, hydrated with distilled water, and stained with Masson trichrome method. Perivascular fibrosis around the vascular tissues was defined as the area of perivascular fibrosis divided by the area of the vascular wall as perivascular fibrosis ratio (PFR) as described previously.^30^, ^31^

### 2.6 Real‐time polymerase chain reaction measurements

Total RNA was extracted from left and right ventricle using RNA Tissue Mini Kit (Qiagen, Hilden, Germany) according to the manufacturer's instructions. Reverse transcription and cDNA synthesis were accomplished using PrimeScript RT Master Mix Perfect Real Time (Takara, Kusatsu, Shiga, Japan). Real‐time polymerase chain reaction was performed to detect the expression of various cytokines by QuantiFast SYBR Green PCR Kit (Qiagen) and Bio‐Rad CFX96 (Bio‐Rad, Hercules, CA, USA) according to the manufacturer's instructions. The conditions of amplification reaction were 95°C for 30 seconds, 95°C for 5 seconds, 60°C for 30 seconds, and PCR was done for 40 cycles. PCR primers are shown in Table S1. Relative gene expression was calculated using the 2^−ΔΔCT^ method. Primers sequences are provided in the Supporting Information.

### 2.7 Western blotting

Total proteins were extracted from left and right ventricle, protein concentrations were determined through bicin choninic acid (BCA) method. After electrophoresis (SDS‐PAGE), transmembrane, blocking (milk protein), incubation with primary anti‐TH polyclonal antibody (1:200, ab137869; Abcam), anti‐NPY polyclonal antibody (1:1000, ab137869; Abcam), anti‐GAP43 monoclonal antibody (1:300, ab117265; Abcam), anti‐PGP9.5 monoclonal antibody (1:200, ab8189; Abcam), anti‐collagen I polyclonal antibody (1:500, GTX20292; GeneTex), anti‐collagen III polyclonal antibody (1:500, NB600‐594; Novusbio, Littleton, CO, USA), and secondary antibody (Goat‐anti‐rabbit, Goat‐anti‐mouse; KPL, Milford, MA, USA), and colouration, immunoreactive bands were obtained. Then the images were captured, and semi‐quantitatively analysed by Quantity One (Bio‐Rad).

### 2.8 Statistical analysis

All data were presented as mean ± SD and assessed by one‐way ANOVA among the groups. The data were analyzed by Tukey′s post hoc test or Games‐Howell test to test the difference between the means of various groups and *P *<* *0.05 was considered as statistically significant (SPSS Statistics 21.0 and GraphPad Prism 5.0).

## 3 RESULTS

### 3.1 Survival

All seven rats assigned to sham operation survived to the study end. 17 out of 18 rats underwent AV operation survived, one rat died because of massive bleeding after AV fistula. Seven rats assigned as aortocaval shunt group (AV) and survived to the study end. Ten rats received additional bilateral SGX operation (AD), one rat died immediately after stellate ganglionectomy surgery, one moribund rat was killed under deep anaesthesia before echocardiography measurement in AD group because of breathing difficulty, eight rats survived to the study end. The data from seven rats in S and AV group, eight rats in AD group were analysed.

### 3.2 Impact of SGX on neurohormones expression in chronic volume overload rats

As shown in Figure 1B, the protein expression of TH, NPY, GAP43 and PGP9.5 in RV was significantly up‐regulated in AV group (vs S group) (*P < *0.01), protein GAP43 and PGP9.5 were significantly down‐regulated in AD group (vs AV group) (*P < *0.05, *P < *0.01). The immunohistochemistry examinations showed that TH, NPY, GAP43 (*P < *0.01) and PGP9.5 (*P < *0.05) expressions were significantly up‐regulated in AV group (vs S group), GAP43 was down‐regulated in AD group (vs AV group) (*P < *0.01) in the RV (Figure 1C).

**Figure 2:**
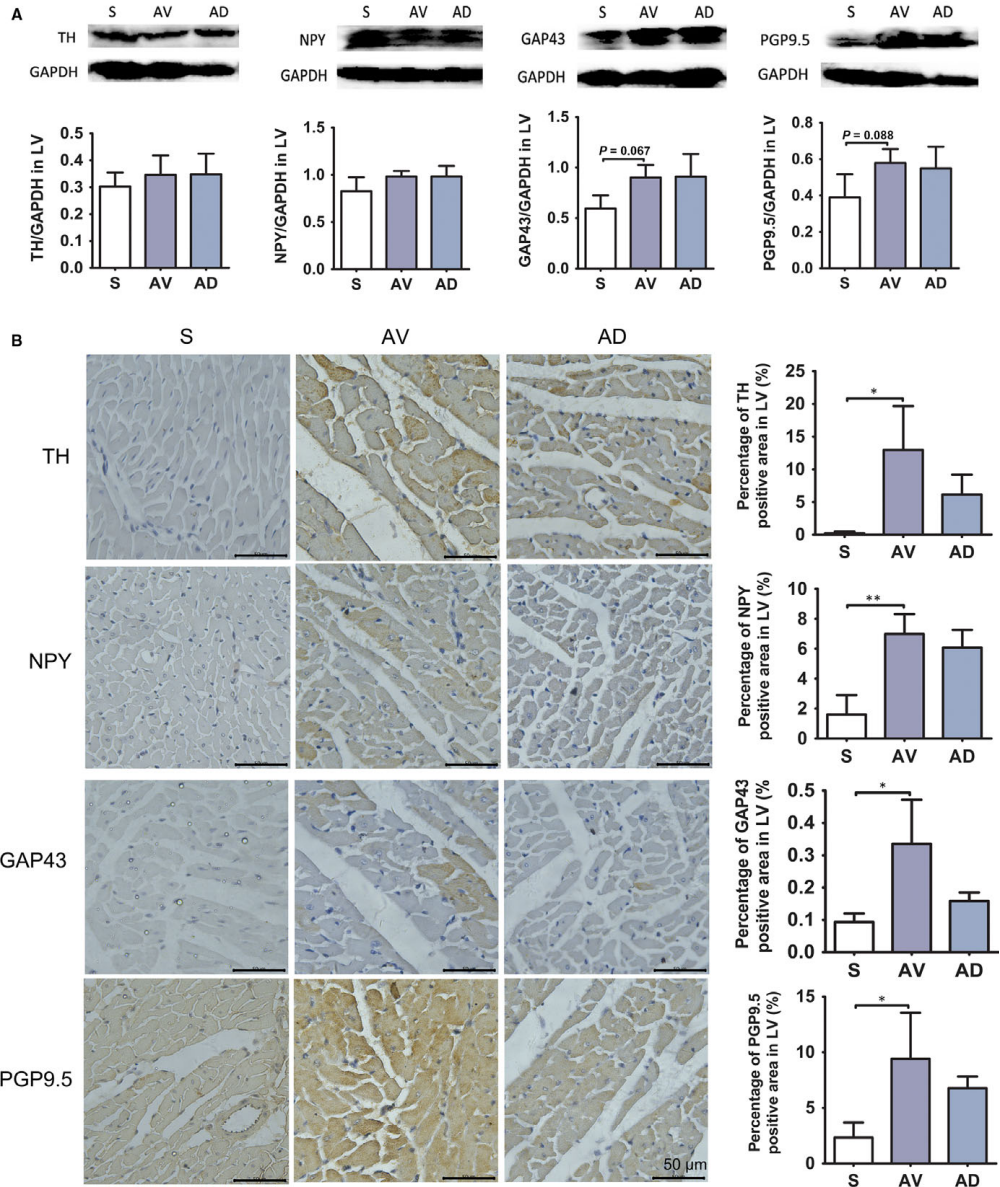
A, Protein expression of sympathetic neurohormones in LV. Protein expression of GAP43 and PGP9.5 tended to be higher in AV group compared to S group in LV (*P *=* *0.067*, P *=* *0.088). (S, AV and AD, n = 4). B, Immunohistochemistry staining of sympathetic neurohormones in LV. The protein expression of NPY (**P <* *0.01), TH, GAP43 and PGP9.5 (*P <* *0.05) were significantly increased in AV group compared to S group in LV (×400). (S, AV and AD, n = 3)

As shown in Figure 2A, protein expression of GAP43 and PGP9.5 tended to be higher in AV group compared to S group in LV (*P *=* *0.067*, P *=* *0.088). Immunohistochemistry results showed that protein expression of TH, GAP43 and PGP9.5, and NPY was significantly up‐regulated in AV group as compared to S group in LV. Above changes were not significantly affected by SGX (Figure 2B).

### 3.3 Impact of SGX on cardiac remodelling and function

**Figure 3:**
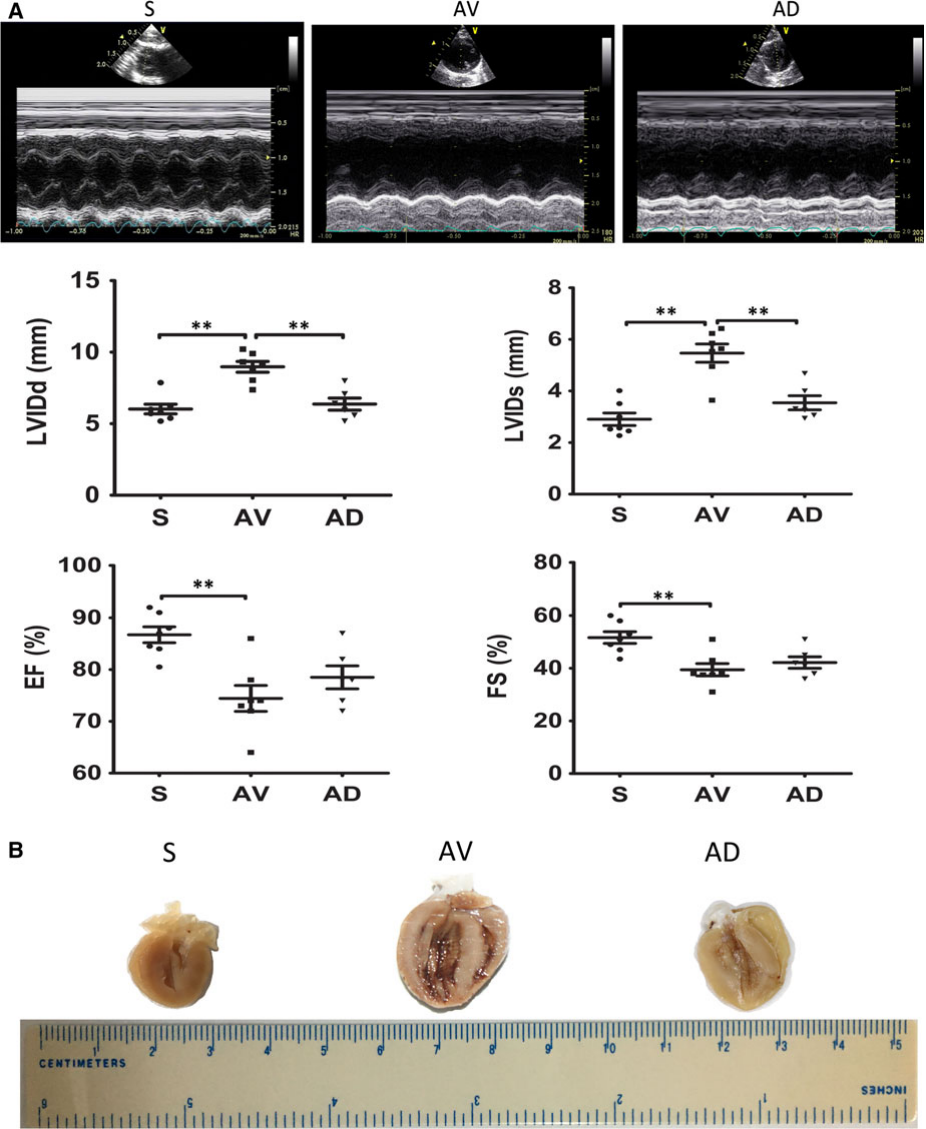
A, Echocardiography results. (a) LVIDd, LV internal diameter at end‐diastole; (b) LVIDs, LV internal diameter at end‐systole; (c) EF, ejection fraction; (d) FS, fractional shortening (**P <* *0.01). (S, n = 7; AV, n = 7; AD, n = 8). B, Gross heart morphology. Ventricular enlargement and cardiac hypertrophy in the AV and AD group. (S, AV and AD, n = 3)

Echocardiography examination showed that LVIDd and LVIDs were significantly increased in AV group (vs S group) (*P *<* *0.01), which were significantly reduced in AD group (*P *<* *0.01 vs AV). EF and FS were significantly reduced in AV group (*P *<* *0.01 vs S), which tended to be higher in AD group compared to AV group (Figure 3A). As expected, heart enlargement, and RV and LV cavity dilation as well as hypertrophied wall thickness were observed in the vertically cut hearts of the AV group and these changes were less apparent in AD group (Figure 3B). Bodyweight was similar among groups, heart, and RV and LV weights were significantly increased in AV group than in S group, which were significantly reduced in AD group (Table 1).

**Table 1:** Bodyweight and heart weight, RV and LV weights

	S (n = 4)	AV (n = 4)	AD (n = 5)
BW (g)	428 ± 37	436 ± 28	402 ± 15
HW (mg)	1422 ± 116	2276 ± 62*	1548 ± 197†
HW/BW (mg/g)	3.34 ± 0.39	5.23 ± 0.24*	3.85 ± 0.46†
RVW (mg)	286 ± 41	536 ± 23*	274 ± 69†
RVW/BW (mg/g)	0.67 ± 0.09	1.24 ± 0.13*	0.68 ± 0.16†
LVW (mg)	1001 ± 85	1490 ± 86*	1097 ± 62†
LVW/BW (mg/g)	2.35 ± 0.28	3.42 ± 0.08*	2.73 ± 0.18†

Mean ± SD.

BW, Bodyweight; HW, Heart weight; RVW, right ventricular weight; LVW, LV weight.

*P <* *0.01 vs S group, ^†^ *P *<* *0.01 vs AV group.

### 3.4 Impact of SGX on cellular remodelling and myocardial hypertrophy markers

**Figure 4:**
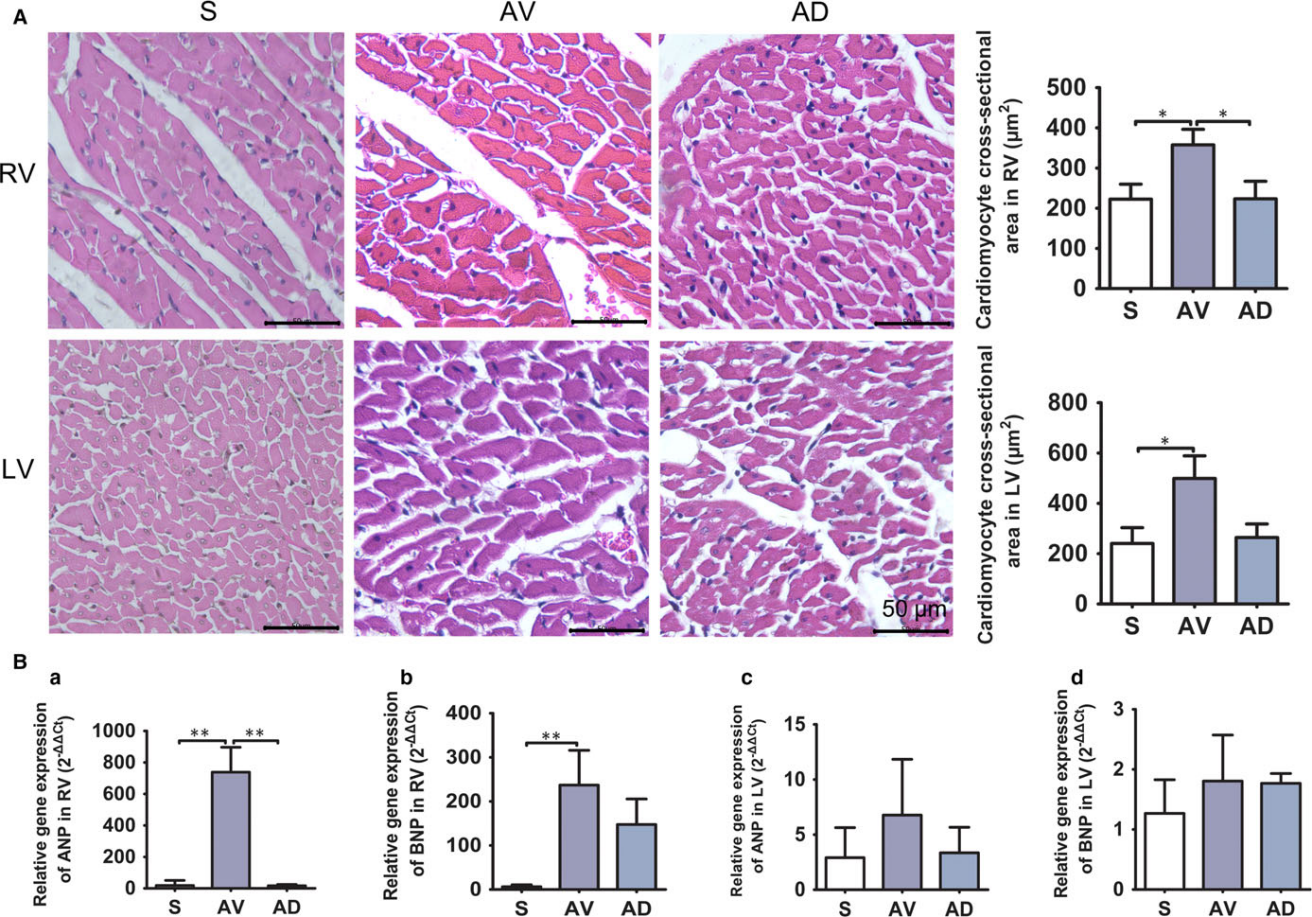
A, Cardiomyocyte size measured on HE stained sections. The cardiomyocyte cross‐sectional area of RV and LV enlarged in AV group compared to S group and was significantly smaller in RV of AD group (*P <* *0.05, ×400). (S, AV and AD, n = 3). B, mRNA expressions of myocardial ANP and BNP in right and left ventricle. mRNA expression of ANP and BNP in RV was significantly up‐regulated in AV group compared to S group, ANP expression was significantly down‐regulated in AD group compared to AV group in RV (a and b, **P <* *0.01), mRNA expression of ANP and BNP in LV was similar among groups (c and d). Quantification graph are shown from triplicate experiments. (S, AV and AD, n = 4)

HE results demonstrated that cardiomyocyte cross‐sectional area in both RV and LV were significantly larger in AV group than in S group (*P *<* *0.05). The cardiomyocyte cross‐sectional area of RV was significantly smaller in AD group than those in AV group (*P *<* *0.05) (Figure 4A). Meanwhile, myocardial mRNA expressions of ANP and BNP, known as myocardial hypertrophy markers,^32^ were significantly up‐regulated in RV of AV group (*P *<* *0.01 vs S), mRNA expression of ANP in RV was significantly down‐regulated in AD group compared to AV group, while ANP and BNP expressions were similar among groups in LV (Figure 4B). We also observed the perivascular coronary fibrosis and found that the perivascular fibrosis ratio was similar among groups (Figure S1).

### 3.5 Impact of SGX on myocardial extracellular matrix and fibrosis

**Figure 5:**
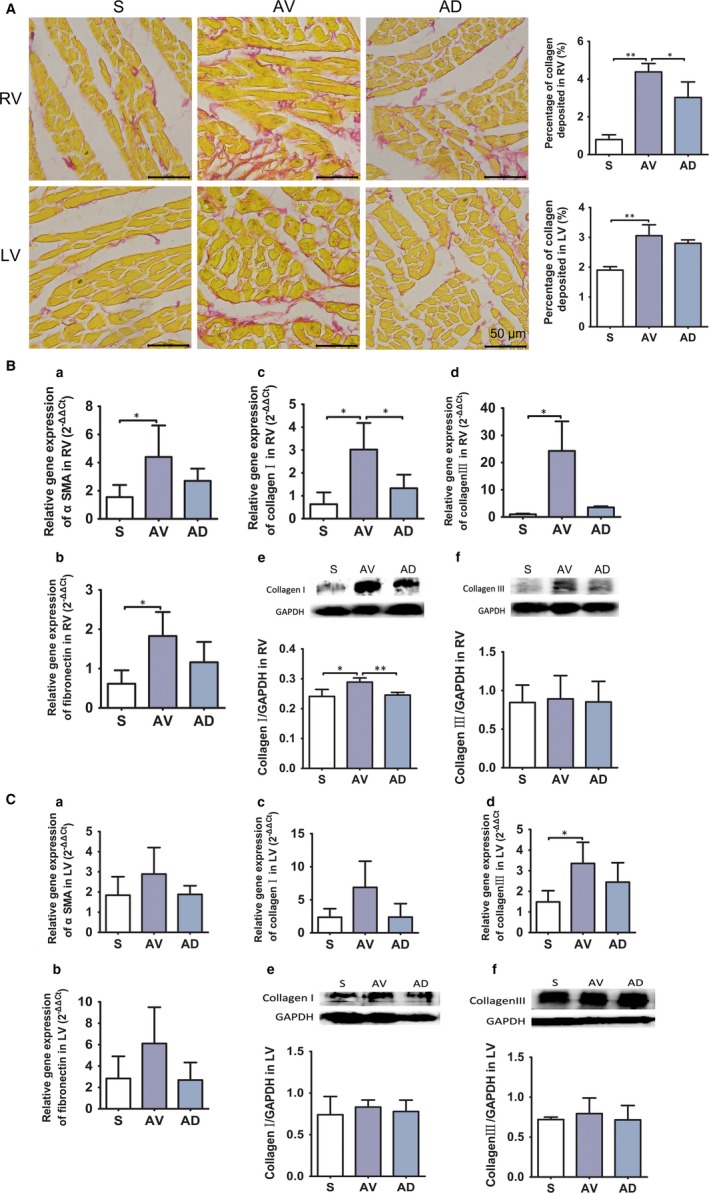
A, Percentage of collagen deposited in myocardial extracellular matrix. Percentage of collagen deposited in myocardial extracellular matrix in RV and LV determined on Sirius red stained sections (×400) (*P <* *0.05,**P <* *0.01). (S, AV and AD, n = 3). B, Myocardial fibrosis cytokines expression in RV. mRNA expression of ɑ‐SMA, fibronectin, collagen I and III was increased in RV of AV group and mRNA expression of collagen I was down‐regulated in AD group (a,b,c and d, *P <* *0.05). Protein expression of collagen I was up‐regulated in AV group and down‐regulated in AD group in RV (e, *P <* *0.05, **P <* *0.01), while protein expression of collagen III in RV was similar among groups (f). Quantification graph are shown from triplicate experiments. (S, AV and AD, n = 4). C, Myocardial fibrosis cytokines expression in LV. mRNA expression of collagen III was increased in LV of AV group (d, *P <* *0.05). Myocardial fibrosis protein expression in LV was similar in various groups in LV (e and f). Quantification graph are shown from triplicate experiments. (S, AV and AD, n = 4)

Results from Sirius red stained heart sample showed that the percentage of collagen deposited was significantly increased in RV and LV in rats from AV group (*P *<* *0.01 vs S group), which was significantly reduced in AD group in RV (*P *<* *0.05 vs AV group) (Figure 5A). mRNA expression of markers of fibrosis like ɑ‐SMA, fibronectin, collagen I, and collagen III in RV were significantly up‐regulated in AV group compared to S group (*P *<* *0.05), mRNA expression of collagen I in RV was significantly down‐regulated in AD group compared to AV group. Protein expression of collagen I was significantly up‐regulated in AV group (*P *<* *0.05) and down‐regulated in AD group (*P *<* *0.01), while protein expression of collagen III was similar among groups in RV (Figure 5B). mRNA expression of collagen III was up‐regulated in AV group (*P *<* *0.05 vs S group) and not affected by SGX, and the rest fibrosis markers were similar among groups in LV (Figure 5C).

### 3.6 Plasma Ang II level and myocardial Ang II and aldosterone expression

**Figure 6:**
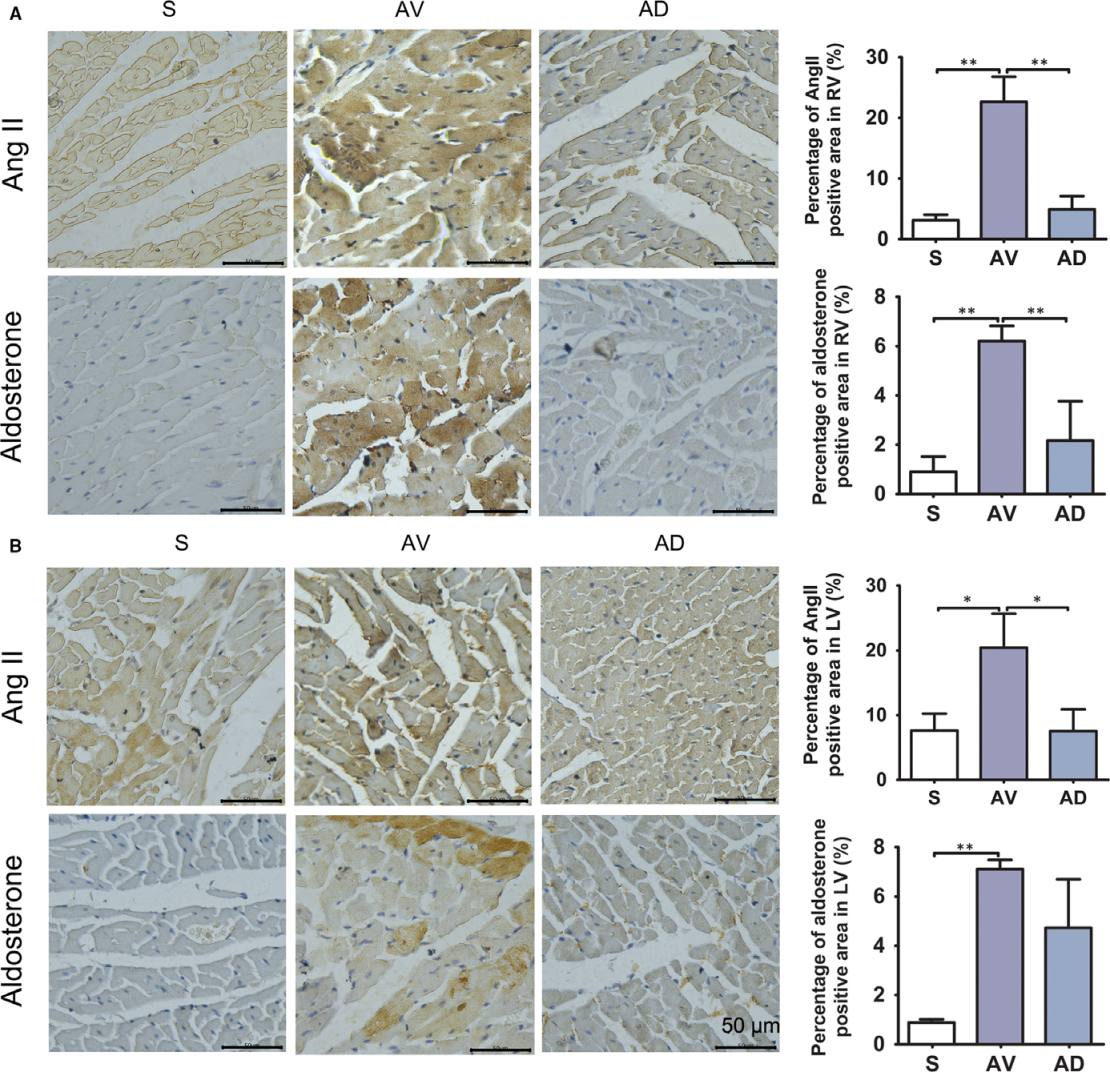
Immunohistochemistry staining of Ang II and aldosterone in RV and LV. Protein expression of Ang II and aldosterone was increased in AV group in RV and LV, and down‐regulated in AD group in RV (*P <* *0.05, **P <* *0.01, ×400). Protein expression of Ang II was also down‐regulated in AD group in LV. (S, AV and AD, n = 3)

Plasma Ang II was similar among groups (S, 0.5270 ± 0.0805 pg/mL; AV, 0.6350 ± 0.0160 pg/mL; AD, 0.5858 ± 0.0599 pg/mL). Myocardial protein expression of Ang II and aldosterone was increased in RV of AV group compared to S group (*P *<* *0.01), which were significantly down‐regulated in AD group (*P *<* *0.01 vs AV group) in RV (Figure 6A). Protein expression of Ang II and aldosterone was increased in LV of AV group compared to S group (*P *<* *0.05, *P *<* *0.01), protein expression of Ang II was down‐regulated in AD group in LV (*P *<* *0.05) (Figure 6B).

## 4 DISCUSSION

Sustained increased sympathetic neurohormones can lead to secondary heart damage after the initial insult.^33^ In this study, chronic volume overload was applied to rats for 12 weeks and sympathetic activation and cardiac remodelling were demonstrated in this model. Our results revealed that “partial cardiac denervation” achieved by bilateral SGX could attenuate myocardial remodelling and fibrosis, and these effects were associated with reduced myocardial sympathetic neurohormones expression, especially in the right ventricle. To our best knowledge, this is the first experimental study reporting on the effect of bilateral SGX and related mechanisms in this chronic volume overload rat model.

### 4.1 Cardiac remodelling and sympathetic activation in rats with chronic volume overload: is there a difference between the right and left ventricle?

AV usually serves as a classical model for volume overload induced chronic heart failure. The major pathological features of this model include the circulating volume overload and aggravated cardiac remodelling, including myocardium hypertrophy and fibrosis.^25^, ^34^, ^35^ Present study demonstrated aggravated cardiac remodelling and increased cardiac sympathetic expression in rats post 12 weeks AV stimulation as shown by increased heart weight, enlarged cardiomyocyte size, enhanced extracellular fibrosis of the heart, and up‐regulated myocardial mRNA expression of ANP and BNP. Chronic volume overload not only initiated the process of histologically detectable cardiac remodelling, but also significantly elevated the level of cardiac sympathetic neurohormones indicated by up‐regulated myocardial expression of TH, GAP43, PGP9.5, and NPY. These data thus collectively indicate that sympathetic activation is a major pathological feature in this model. In this AV model of rats, RV and LV faced various loading burden. Previous experimental studies demonstrated that, besides to deal with the same increased circulating volume in RV and LV, RV also faced an increased pressure load from the increased pulmonary systolic pressure in this model.^36^, ^37^ This might partly explain the stronger sympathetic activation observed in the RV than in the LV in this model.

### 4.2 Impact of SGX on cardiac remodelling and neurohormones

Heart receives multiple sympathetic dominance, which includes the superior cervical nerve, the middle cervical, and the stellate nerve. The selective removal of the stellate ganglion could be viewed as “partial cardiac denervation” and capable of decreasing sympathetic activity of the heart partly. Cardiac sympathectomy can be achieved by direct surgical approaches^38^ or by video‐assisted thoracoscopic surgery.^39^ Multiple procedures are reported to achieve the cardiac sympathectomy clinically. Schneider et al dissected the left stellate ganglion in two halves, separated the first thoracic part from the inferior cervical part. The cephalic portion of the stellate ganglion was preserved to prevent Horner syndrome. Then the thoracic ganglia T2–T4/T5 and their associated rami communicantes were removed.^38^ Kopecky et al reported successful experience of bilateral video‐assisted thoracoscopic surgery with T1‐T4 sympathectomy for treatment of refractory ventricular tachycardia.^24^ Our initial goal is to achieve a “partial cardiac denervation” in the chronic volume overload model of rats to preserve some functional responses of the sympathetic nerve despite the bilateral SGX. Previously, Gan et al performed the cardiac sympathetic denervation through bilateral SGX plus transection of T1‐T4 sympathetic rami in rats with experimental myocardial infarction.^40^ As the SGX surgery denervated heart partly, it did not induce denervation supersensitivity.^41^ Previous studies have confirmed the pathophysiology of heart failure resulted in neurohormonal activation and autonomic imbalance with increase in sympathetic activity and withdrawal of vagal activity.^42^ Peter J. Schwartz showed that SGX increased cardiac vagal efferent activity.^43^, ^44^ Based on the close correlation between sympathetic neurohormonal activity and cardiac remodelling in this model, we assumed that secondary cardiac damage induced by excessive sympathetic activation could be partly blocked by bilateral SGX despite the persistent existence of volume overload in this model. Accordingly, our study results demonstrated that cardiac remodelling attenuation was achieved by this “partial cardiac denervation” proposal. Compared to AV group, the sympathetic hormones marker including TH, GAP43, PGP9.5, and NPY were significantly down‐regulated in AD group, especially in the RV, indicating reduced cardiac sympathetic activity post‐bilateral SGX (Figures 1 and 2). Besides attenuated myocardial remodelling, the myocardial fibrosis was also reduced by bilateral SGX, suggesting the regulation of cardiac sympathetic neurohormone activity could also beneficially affect the cardiac matrix deposition. Previous study showed that vagal nerve activity modulation also affected the cardiac remodelling,^45^ future studies are warranted to test the impact of SGX on vagal nerve activity and remodelling in this study.

It is to note that the influence of bilateral SGX was stronger in the RV than in the LV in this model. Sympathetic neurohormones level reduction post‐bilateral SGX was more significant in RV than in LV in this model. The underlying reasons remain elusive. Besides the chronic volume overload for both RV and LV, RV experienced also the extra burden, that is, the increased systolic pulmonary pressure. It remains unknown if the more significant effect on the neurohormone levels could be explained by the more significantly up‐regulated neurohormone levels in the RV as compared to LV in this model. Future studies are warranted to get more evidence to fairly explain the observed findings. Both cardiac remodelling attenuation and sympathetic neurohormones inactivation post bilateral SGX were more significant in RV than in LV, these results suited well with our finding in that the sympathetic activation and remodeling changes were more significant in RV than in LV because of the extra burden on the RV by the increased systolic pulmonary pressure. Our results thus hint that the RV suffered more serious damage and also benefited more from bilateral SGX in the case of unchanged chronic volume loading in this model. “Partial cardiac denervation” might thus not only be useful for treating patients with VF and VT, but also serve as a novel strategy for treating patients with chronic heart failure because of volume overload.

### 4.3 Impact of SGX on renin‐angiotensin‐aldosterone system and sympathetic nervous system

SNS and RAAS are the two major neurohormonal systems, which play crucial role in both physiological and pathological conditions. Experimental and clinical data^1^ showed that the progression of heart failure was the result of activation of many bioactive factors, including mediators of SNS and RAAS. Ang II and aldosterone levels, which characterize the activation of the RAAS. In the case of heart failure, the early compensatory mechanism was the sympathetic nervous system activation, while RAAS activated relatively late. Ang II could stimulate the production of aldosterone by enhancing sympathetic released adrenaline and adrenal cortical globular bands, aldosterone can further activate the SNS.^46^ Mounting evidence indicate that RAAS is actively involved in the pathogenesis of heart failure.^47^, ^48^ The interaction between SNS and RAAS in the case of heart failure is schematically presented in the Figure S2.

Our study shows that bilateral SGX also reduced the activity of RAAS in cardiac tissue, as shown by reduced expression of Ang II and aldosterone in the AD group as compared to AV group (Figure 6). These results suggested that lowering SNS activity by bilateral SGX could also effectively affect the RAAS axis and reduced RAAS activity. Thus, bilateral SGX could affect both SNS and RAAS systems, changes on both systems might contribute to the observed beneficial effects on the cardiac remodelling and fibrosis in this model.

### 4.4 Study limitations

There are several study limitations of our experiments.

1. The animal numbers were small and the time course of partial cardiac sympathetic denervation was not observed in this model, cautions are therefore needed on interpretation of the current study results. Studies are needed to explore the impact of SGX in animal models with long‐standing (more time points) and overt cardiac remodelling to observe if SGX strategy might be also beneficial or not to reverse the preexisting cardiac remodelling because of chronic volume or pressure overload.
2. The changes of sympathetic neurohormones post‐bilateral SGX was multiple and also affected vagal nerve activity changes. Present study focused on the myocardial sympathetic mediator changes in the setting of chronic volume overload and SGX, future studies are warranted to explore the impact of SGX on vagal nervous system and the autonomic imbalance between sympathetic activity and vagal activity in the setting of chronic volume overload and SGX.
3. In this study, we observed considerable benefits of the “partial cardiac denervation” through bilateral SGX in this model, however, the potential clinical advantage of the bilateral SGX over the more and more extensive denervation remains unknown now, future studies are warranted to compare the effects of bilateral SGX vs more extensive cardiac denervation in this model.
4. It is to note that presentation of data of the Northern blots for the RNA expression of ANP and BNP in RV and LV tissues might be helpful to reflect the disease process and the effects of bilateral SGX in this model, because of technical limitations of our laboratory, these data were not determined and further studies are needed to explore related issues. We have also no data on the ratio of the newly synthetized collagen type I to degraded/denaturated collagen I, which is another study limitation of this experimental study.

## 5 CONCLUSION

In conclusion, our study indicates that “partial cardiac denervation” by bilateral SGX could attenuate cardiac remodelling and fibrosis by reducing myocardial sympathetic neurohormones expression in this rat model of chronic volume overload, these beneficial effects are at least partly mediated by reduced myocardial sympathetic and RAAS activities. Our study provides initial experimental evidence and potential mechanism of bilateral SGX for the treatment of heart failure induced by chronic volume overload.

## CONFLICT OF INTERESTS

The authors confirm that there are no conflicts of interest.
