# Supplementary material for: Bilateral sympathetic stellate ganglionectomy attenuates myocardial remodelling and fibrosis in a rat model of chronic volume overload
Source: J Cell Mol Med. 2018 Nov 8;23(2):1001–13. doi: 10.1111/jcmm.14000 (PMC6349216; doi:10.1111/jcmm.14000)
Supplement: Supplementary file 1 [file JCMM-23-1001-s001.pdf]

Supplementary Figure 1

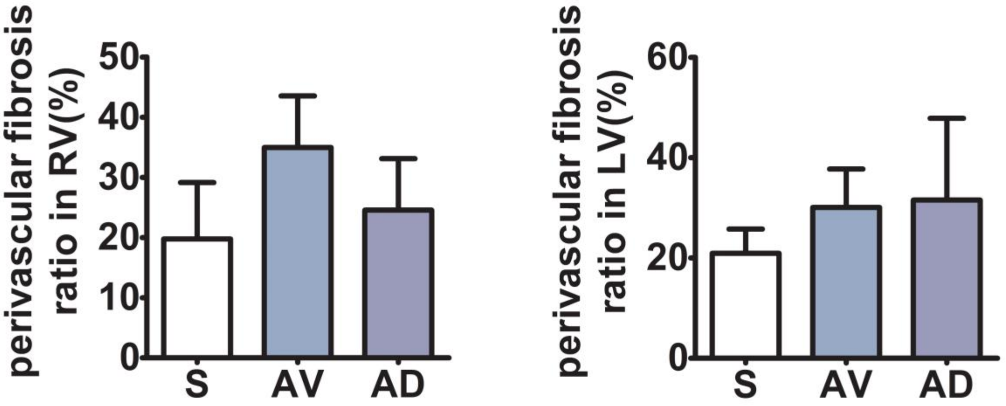

Supplementary Figure 1 Perivascular fibrosis ratio in right and left ventricle

The perivascular fibrosis ratio was similar among groups in right and left ventricle. (S, AV and AD, n=3)

Supplementary Figure 2

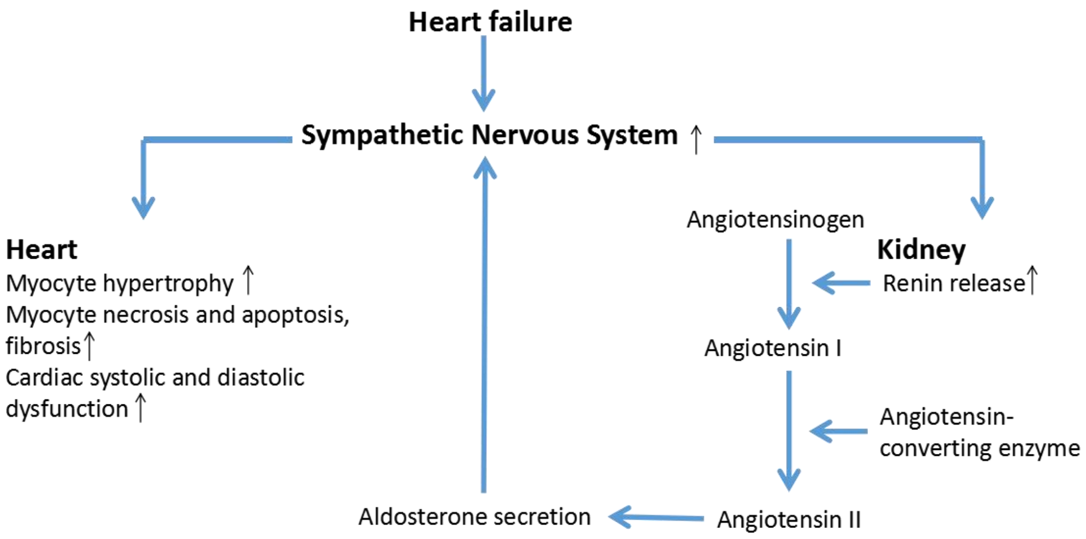

Supplementary Figure 2 The interaction between SNS and RAAS in the case of heart failure.

Supplementary Figure 3

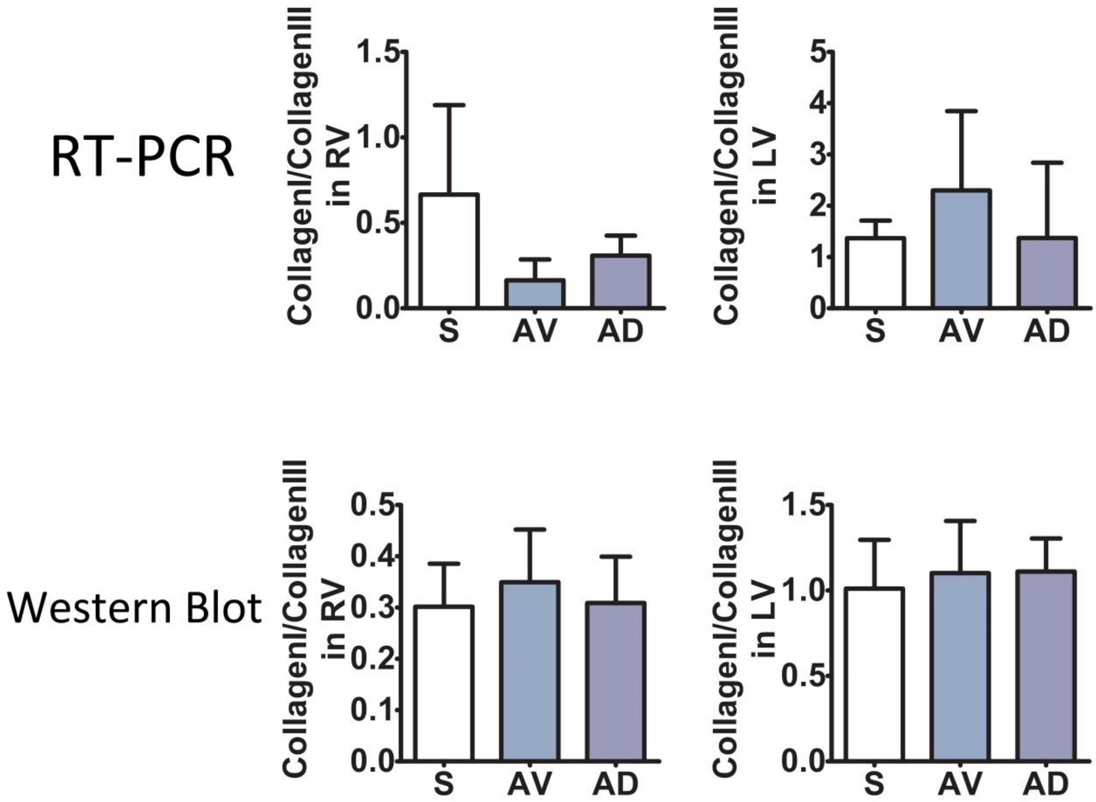

Supplementary Figure 3 The ratio of collagen I to collagen III in right and left ventricle

The ratio of collagen I to collagen III was similar among groups in right and left ventricle. (S, AV and AD, n=4)
