# Supplementary material for: Bilateral sympathetic stellate ganglionectomy attenuates myocardial remodelling and fibrosis in a rat model of chronic volume overload
Source: J Cell Mol Med. 2018 Nov 8;23(2):1001–13. doi: 10.1111/jcmm.14000 (PMC6349216; doi:10.1111/jcmm.14000)
Supplement: Supplementary file 2 [file JCMM-23-1001-s002.pdf]

**Supplementary Table 1 RT-PCR Forward/Reverse (F/R) Primers Sequences**

| Forward/Reverse | Sequence (5'-3') |
|---|---|
| GAPDH | F: 5′ -CGCTAACATCAAATGGGGTG-3′ |
| | R:5′ -TTGCTGACAATCTTGAGGGAG-3′ |
| ANP | F: 5′ -CTTCGGTACCGGAAGCTGTT-3′ |
| | R: 5′ -GGGAAGTCAACCCGTCTCAG-3′ |
| BNP | F:5′-GCAGCTTGAACTATGTGCCATC-3′ |
| | R: 5′ -CTCAAAGGACCAAGGCCCTAC-3′ |
| ɑ-SMA | F:5′-AGCATCCGACCTTGCTAACG-3′ |
| | R: 5′ -TGAGTCACGCCATCTCCAGAG-3′ |
| fibronectin | F:5′-GAGGCACAAGGTCCGAGAAGAG-3′ |
| | R:5′-GAAACCGTGTAAGGGTCAAAGCA-3′ |
| Collagen I | F:5′-TTTAATGGATAGGGACTTGTGTGAA-3′ |
| | R:5′-GAGAGAGAGAGAAGCTGAGGGTAGG-3′ |
| Collagen III | F:5′-GGTTTGGAGAATCTATGAATGGTGG-3′ |
| | R:5′-GCTGGAAAGAAGTCTGAGGAAGG-3′ |

GAPDH, glycerinaldehyd-3-phosphat-dehydrogenase; ANP, atrial natriuretic peptide; BNP, brain natriuretic peptide; ɑ-SMA, α-smooth muscle actin.
